# RETRACTION: Prevalence of Surgical Site Infection and Risk Factors in Patients after Foot and Ankle Surgery: A Systematic Review and Meta‐Analysis

**DOI:** 10.1111/iwj.70291

**Published:** 2025-03-03

## Abstract

**RETRACTION:** ChengJ. , ZhangL. , ZhangJ. , AsadiK. , and FarzanR. , “Prevalence of Surgical Site Infection and Risk Factors in Patients after Foot and Ankle Surgery: A Systematic Review and Meta‐Analysis,” International Wound Journal 21, no. 1 (2024): e14350, 10.1111/iwj.14350.37606302 PMC10781588

The above article, published online on 22 August 2023, in Wiley Online Library (http://onlinelibrary.wiley.com/), has been retracted by agreement between the journal Editor in Chief, Professor Keith Harding; and John Wiley & Sons Ltd. Following an investigation by the publisher, all parties have concluded that this article was accepted solely on the basis of a compromised peer review process. In addition, the investigation found significant textual overlap between the methods and discussions sections of this article and another article by some of the same authors [Asadi et al. 2023 [https://doi.org/10.1111/iwj.14300]). The editors have therefore decided to retract the article. The authors disagree with the retraction.

**RETRACTION:** J. Cheng , L. Zhang , J. Zhang , K. Asadi , and R. Farzan , “Prevalence of Surgical Site Infection and Risk Factors in Patients after Foot and Ankle Surgery: A Systematic Review and Meta‐Analysis,” International Wound Journal 21, no. 1 (2024): e14350, 10.1111/iwj.14350.37606302 PMC10781588

The above article, published online on 22 August 2023, in Wiley Online Library (http://onlinelibrary.wiley.com/), has been retracted by agreement between the journal Editor in Chief, Professor Keith Harding; and John Wiley & Sons Ltd. Following an investigation by the publisher, all parties have concluded that this article was accepted solely on the basis of a compromised peer review process. In addition, the investigation found significant textual overlap between the methods and discussions sections of this article and another article by some of the same authors [Asadi et al. 2023 [https://doi.org/10.1111/iwj.14300]). The editors have therefore decided to retract the article. The authors disagree with the retraction.

